# Novel Insect Florivory Strategy Initiates Autogamy in Unopened Allogamous Flowers

**DOI:** 10.1038/s41598-018-35191-z

**Published:** 2018-11-20

**Authors:** N. K. Hillier, E. Evans, R. C. Evans

**Affiliations:** Acadia University, Wolfville, NS Canada

## Abstract

Insects may influence plant development via pollination, galling, and a range of herbivorous interactions, including florivory. Here, we report a novel form of insect-plant interaction in the form of florivory-initiated autogamy. *Mompha capella* larvae, feeding on petal bases of *Crocanthemum canadense* before flowers open, while providing no benefit to the plant, cause autogamy and subsequent seed and fruit development. This interaction provides a clear benefit to the florivore because it enters the developing fruit and consumes most seeds; however, surviving seeds are viable. This novel interaction is discussed with respect to the dimorphic cleistogamous reproduction employed by this plant species. Moreover, this represents a previously undocumented insect-plant interaction in the form of a florivory-initiated pollination.

## Introduction

Plant-insect interactions are widely studied to test evolutionary and ecological theory; however, scientists have only begun to appreciate the diversity of such interactions and their impact on plant reproductive systems^1–6^. Florivory (consumption of floral structures by herbivores) may negatively impact plants by depleting resource sinks, altering floral display, and reducing nectar production; the latter two indirectly influencing reproduction by affecting pollinator visitation^3^. However, if florivore activity promotes pollination, a mutualism may evolve^7,8^. Such is the case with mutualistic nurseries and pollination in fig wasps (Chalcidoidea), or coevolution of pollination-herbivory observed in Yucca moths (Prodoxidae) and Yucca plants (Asparagaceae: Agavoideae). In each case, cross-pollination accompanies direct feeding by predators on reproductive plant structures^9,10^. Whereas the expectation might be direct fitness losses from herbivory, in these interactions pollination provided by florivores benefits the host’s fitness. Many empirical studies of florivore-induced pollination have focused on florivore-mediated cross-pollination (allogamy), rather than self-pollination (autogamy)^5,8,11^. Here we report florivore-initiated autogamy in Canada Frostweed, *Crocanthemum canadense* L. (Britton).

**Figure 1:**
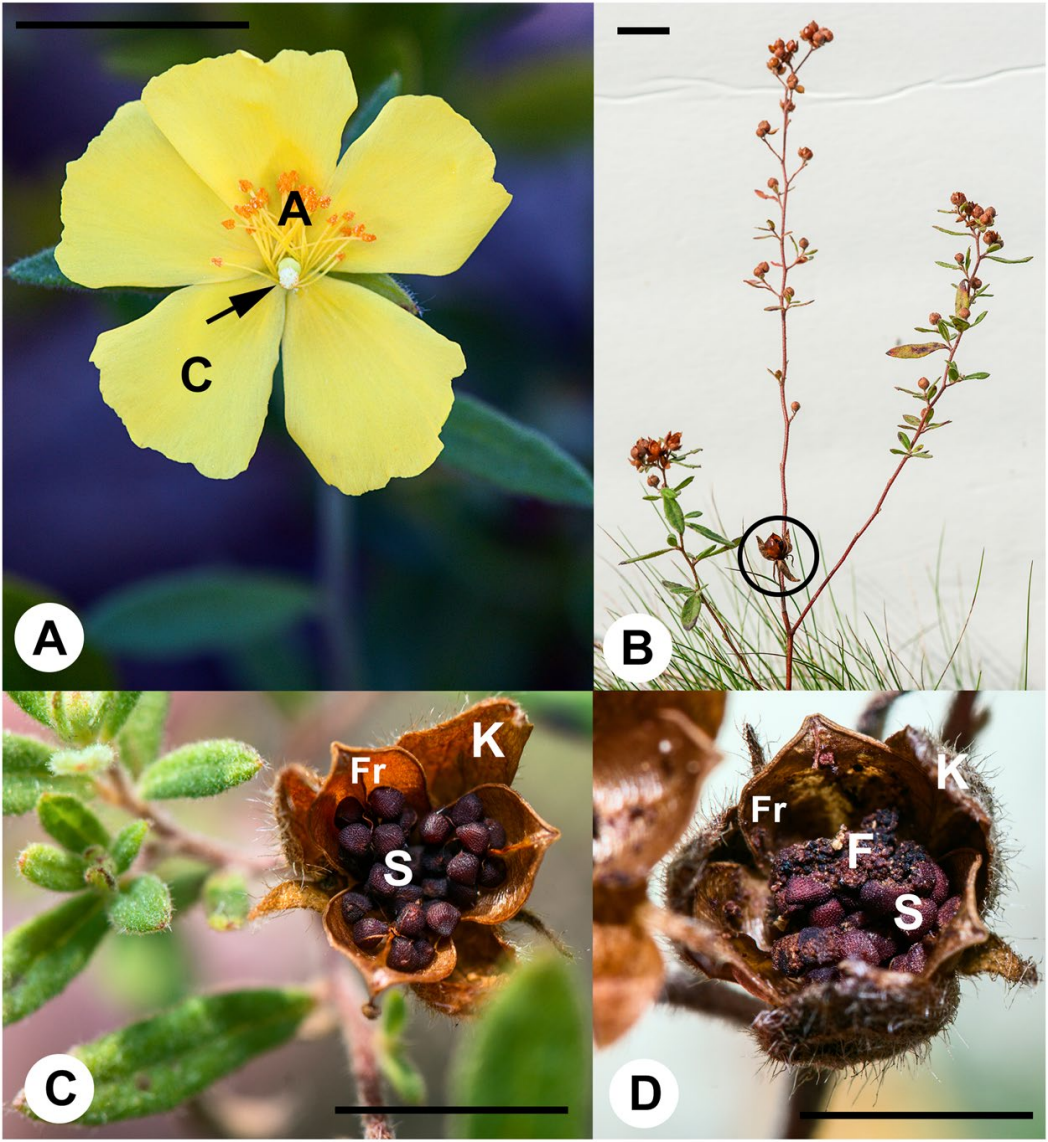
*Crocanthemum canadense* flower and fruit morphology. (**A**) Open chasmogamous flower. (**B**) Late season *C. canadense* plant with chasmogamous fruit (circle) and several lateral, axillary branches covered in multiple cleistogamous flowers and developing fruits. (**C**) Dehiscing chasmogamous fruit with seeds. (**D**) Dehiscing chasmogamous fruit with insect frass and several (partially) intact seeds. Arrow = pistil; A = anther; C = petal; Fr = fruit valve; F = insect frass; K = sepal; S = seeds. Scale bars = 15 mm.

*Crocanthemum canadense* (Cistaceae) is an herbaceous perennial restricted to dry, sandy pine barren ecosystems in eastern North America. *Crocanthemum canadense* is reported in all Eastern US states but Florida, West to Alabama and North to Minnesota (i.e. east of the Mississippi River), as well as Nova Scotia, Quebec and Ontario in Canada. Conservation status for many states is “unknown”, and is listed as “secure” in New York and Virginia, but “vulnerable” to critically imperiled in several states and provinces throughout its distribution (http://explorer.natureserve.org/servlet/NatureServe?searchName=Helianthemum+canadense). With only 5000–5500 plants restricted to reatively few available sand barrens in Nova Scotia, *C*. *canadense* is listed as critically imperiled^12,13^, http://explorer.natureserve.org/servlet/NatureServe?searchName=Helianthemum+canadense]. The reproductive biology of this species is of interest, as it employs dimorphic cleistogamy whereby plants produce chasmogamous (open; Fig. 1A) and cleistogamous (closed; Fig. 1B) flowers^14,15^. Although a number of angiosperms employ cleistogamy as a reproductive biology, relatively few (148 species in 67 genera, 29 families) are classified as dimorphic cleistogamous^15^.

**Figure 2:**
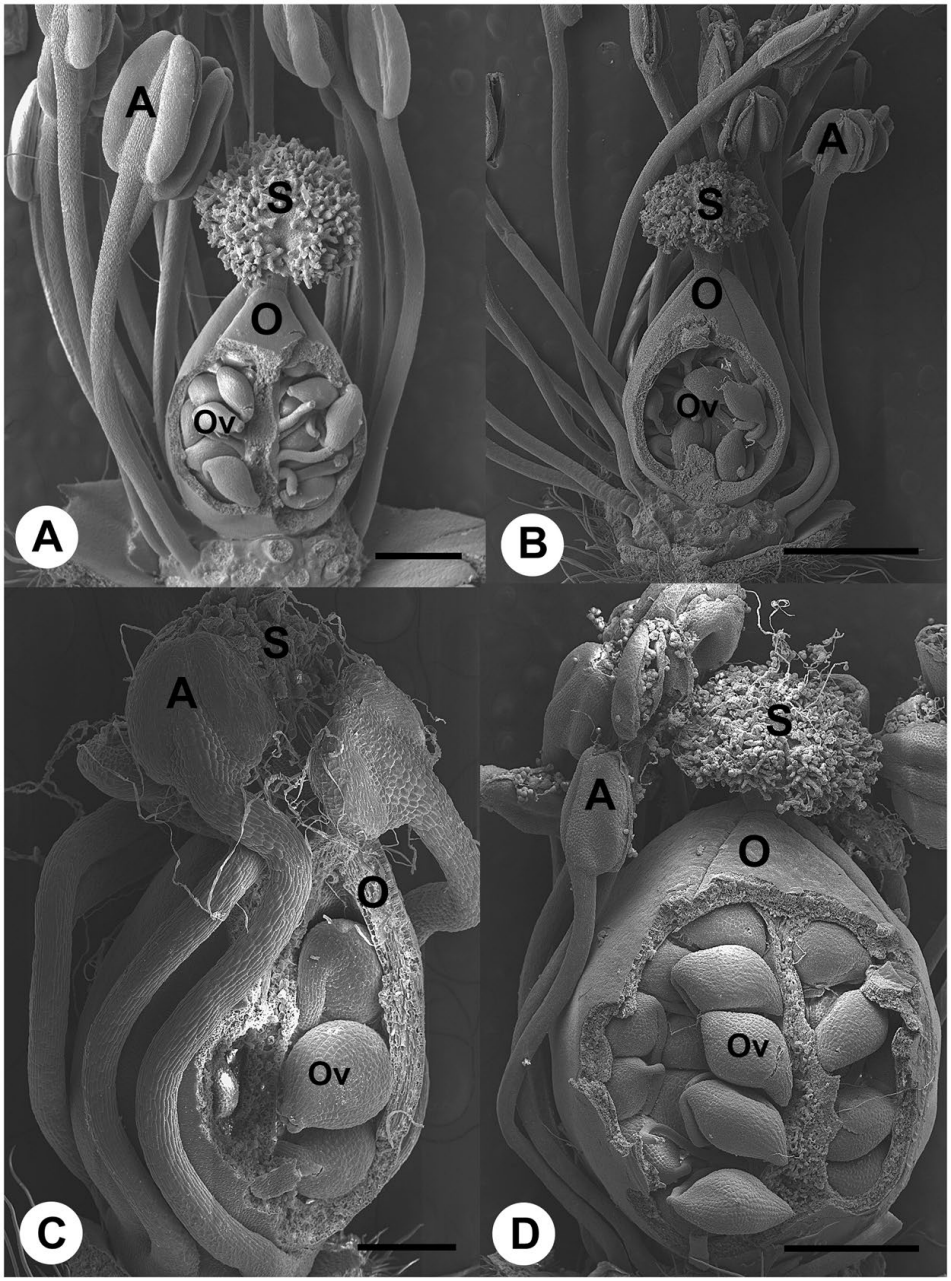
Scanning electron micrographs of reproductive biology in chasmogamous, cleistogamous and florivore induced-pollination flowers of *C. canadense*. (**A**) Partially dissected (sepals and petals removed) pre-anthesis chasmogamous flower. Stamens are erect and anther sacs have yet to dehisce. Stigmatic surface is devoid of pollen. (**B**) Partially dissected (sepals are removed) post-anthesis chasmogamous flower. Anther sacs have dehisced and are devoid of pollen. Stigma is covered with pollen and pollen tubes are visible within the ovary amongst the ovules. (**C**) Cleistogamous flower with sepals removed and partially dissected ovary. Filaments of five stamens are visible in close proximity to the ovary wall. Anther sacs of each stamen are positioned above the stigma and pollen tubes are visible coming from within. (**D**) Dissected chasmogamous flower with petal cone removed to illustrate dehiscing anthers near the stigma. Pollen grains and pollen tubes are visible on the stigma, and pollen tubes are visible within the ovary near the ovules. A = anther; O = ovary Ov = ovule; S = stigma. Scale bar = 500 μm (**A**); 1 mm (**B** and **D**); 200 μm (**C**).

Typically, a single *C*. *canadense* plant produces relatively few showy, yellow-petalled, chasmogamous (open-pollinated) flowers (Table 1; Fig. 1A) in late June through early July that are pollinated by small bees and flies. Pre-anthesis, chasmogamous flowers have five sepals, alternating with five petals, and multiple erect stamens surrounding the pistil (Fig. 2A). Chasmogamous flowers typically open just after sunrise, stamens retract from around the pistil and lay against the petals (Fig. 1A), reducing the likelihood of autogamy through spatial separation of stamens and pistil (herkogamy). Within a few hours petals abscise, and stamens are devoid of pollen, owing to insect-based allogamy. If successfully pollinated, sepals of flowers close over the pistil and provide protection for a developing fruit (Fig. 2B).

**Table 1:** Summary of numbers of flowers, *Mompha* infestation, Seeds per flower, and germination rate.

	A: Mean ± SE Flowers/Plant** (N = 38)	B: Mean ± SE Seeds/Flower** (N = 120)	C: Mean ± SE% Germination***(N = 20 Seeds/Plate)	Estimated Reproductive Potential (A × B × C)
Chasmogamous	1.9 ± 0.14^a^	36.2 ± 1.42^a^	90 ± 2.3 (N = 16 plates)^a^	**65**
Cleistogamous	54.7 ± 5.27^b^	6.5 ± 0.39^b^	39 ± 10.1 (N = 6 plates)^b^	**139**
Infested Chasmogamous (N = 30)*	1.9 ± 0.14	1.8 ± 0.66^c^	95 ± 2.9 (N = 3 plates)^a^	**3.4**

*Flowers collected for flower number, seed number, and germination independent of flowers collected for infestation analysis (see Materials and Methods).

**Unpaired two-tailed T-test with a Welch’s correction, superscript letters denote significantly different means, p < 0.05).

***One-Way ANOVA, Tukey’s Multiple Comparison Test (p < 0.05).

Later in the season (late July through August), the same plant produces significantly more cleistogamous flowers (closed, autogamous) on axillary branches that develop below chasmogamous flowers (Table 1; Fig. 1B). Cleistogamous flowers do not produce petals and never open. Furthermore, they produce only 4–5 stamens that wrap around the developing pistil during early floral organogenesis (compare Fig. 2A versus 2C). When mature, pollen tubes emerge from anthers, onto the stigma, and ultimately into the ovary to fertilize the ovules within (Fig. 2C). Although allogamous chasmogamous flowers produce significantly more seeds per bloom versus cleistogamous flowers (Table 1), total reproductive potential needs to factor the number of each flower type per plant. On average each plant produces 1–3 chasmogamous flowers, and 10–60 cleistogamous flowers in a single growing season. Chasmogamous fruits contain ~40 seeds on average, whereas cleistogamous flowers produce ~8 seeds per fruit^16,17^.

**Figure 3:**
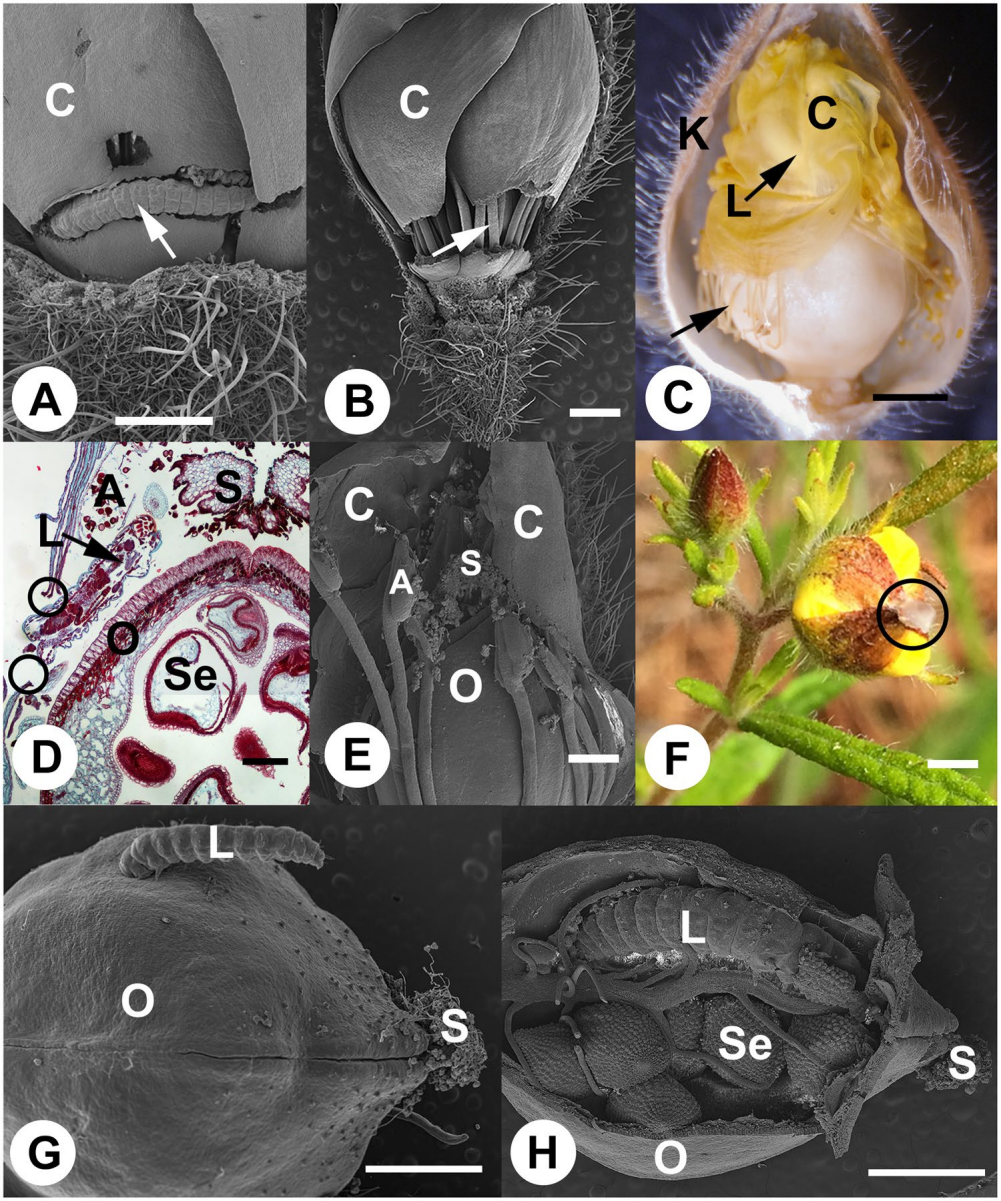
Dissecting microscope, paraffin embedded section and scanning electron micrographs (SEM) of *M. capella* larva-infested pre-anthesis *C. canadense* flowers and developing fruits. (**A**) SEM of flower with two sepals removed to show larva (arrow) chewing through the base of several petals. (**B**) SEM of flower to show that petals are completely severed, but that stamen filaments (arrow) remain intact. (**C**) Image of partially dissected flower with cone of desiccating petals. Larva is visible inside petal cone. Stamen filament separation (arrow) due to early development of ovary into fruit following autogamy. (**D**) Safranin and Fast-Green stained paraffin section showing position of larva inside petal cone. Damage to petals is apparent where larva chewed through petal cone (circles). (**E**) SEM of partially dissected petal cone showing dehisced anthers and pollen on stigmas. (**F**) Example of flower with glue applied to tips of petals to keep them from opening. (**G**) Larva eating an entrance hole into the side of a developing fruit post autogamy. Pollen and pollen tubes are visible on the stigma. (**H**) Partially dissected chasmogamous fruit containing larva and frass. C = petal; K = sepal; L = larva; O = ovary; S = stigma; Se = seed. Scale bar = 200 μm (**G**) 500 μm (**A**, **B** and **E**); 1 mm (**C** and **D**); 5 mm (**F** and **H**).

Our interest in *C*. *canadense* reproductive biology began when Yorke *et al*.^14^ reported that gene flow was absent between populations in Nova Scotia, and within-population genetic variation was lowest of all populations surveyed in northeastern North America. These findings raise questions regarding what factors might be responsible for the occurrence of genetically depauperate populations in Nova Scotia. During comparative analyses of floral development and reproductive biology, we observed open fruits from chasmogamous flowers which completely lacked seeds, and which were filled with insect frass (compare Fig. 1C,D). Subsequent work led to the discovery that florivore-initiated autogamy takes place in unopened chasmogamous flowers invaded by early instar larvae of *Mompha capella* Busck moths (Lepidoptera: Momphidae; Fig. 3A–D)^18^. Although florivory affecting reproductive biology is well documented in many species^3,5,7^, autogamy from florivore damage in pre-anthetic, allogamous flowers has never been reported.

With an understanding of *C*. *canadense* reproductive biology, we next investigated florivore-host relations, hypothesizing that previously observed^14^ reduced genetic variability in Nova Scotia populations may be a result of larvae feeding on seeds in developing allogamous chasmogamous fruit. Ultimately, over half of flowers surveyed were infested with *M. capella* larvae (Table 1).

SEM microscopy evidence showed that larvae were not feeding on seeds produced following allogamy, but that florivory initiated seed production by autogamy in pre-anthesis chasmogamous flowers was occurring in flowers infected with *M*. *capella* larvae. In chemically-fixed material, larvae were observed between sepals and petals of unopened chasmogamous flowers prior to anthesis. After entering a flower, early instar larvae sever petals from the floral receptacle (Fig. 3A,B). As damaged flowers continue developing, petals wither, compacting the stamens within a cone of petal tissue around the distal end of the pistil (Fig. 3C–E). At later stages, larvae were also observed between petals and ovaries consuming petal, anther tissues, and pollen (Fig. 3C,D). Infested flowers never opened and autogamy occurred as drying anthers dehisced atop the stigma (Figs 2D and 3E). Pollen tubes were produced, entering ovaries through the stigma, and fertilizing ovules (Fig. 3E). To our knowledge, this is the first report of such florivore-initiated autogamy.

In order to demonstrate that autogamy was the result of flowers not opening (i.e. anthesis), we glued the petal tips of 14 flowers prior to anthesis (Fig. 3F). Flowers were re-examined after fruit set of neighboring plants in the population. During that time, none of the flowers were observed opening. Thirteen of the fourteen flowers produced fruit autogamously, one flower abscised from the plant. This suggests that the mechanism for autogamy is the result of flowers not opening due to physical manipulation. Physical manipulation of petals, by insect florivory or gluing petal tips, removes the possibility of herkogamy which promotes allogamous fruit production in this species.

Before the fruit matures, larvae bore an entrance hole into the developing fruit wall (Fig. 3G). Once inside the developing fruit, larvae consume most to all of the seeds (see infested chasmogamous seeds/flower, Table 1; Figs 1D and 3H). Seed germination rates from uninfested allogamous fruits and florivory-initiated fruits were not significantly different, but germination rates from autogamous cleistogamous fruits were significantly lower (Table 1).

*Crocanthemum canadense* likely experiences a significant fitness loss due to infestation by *M. capella* affecting its normal reproductive biology. Because the florivore preys upon pre-anthetic, chasmogamous flowers, only 46% (N = 69/150) of chasmogamous flowers remained uninfested and available to typical pollinators. In terms of estimated reproductive potential, a single uninfested plant will produce roughly 65 allogamous, germinating progeny/plant resulting from chasmogamous flowers (Table 1). This is low in comparison to 139 progeny expected from cleistogamous flowers (Table 1). However, when infested, productivity of normal chasmogamous flowers is reduced to 3.4 seeds/plant (Table 1). Furthermore, any progeny resulting from *M. capella*-infestation will have decreased genetic diversity due to autogamy, similar to that expected in seeds from cleistogamous fruits. Fitness reductions from feeding and damage to floral structures in floral nursery systems may be balanced by pollination service, however the florivory described herein may contribute to reduced genetic diversity observed in Nova Scotia populations^7,8,14,19^.

Initiation of autogamy in chasmogamous flowers appears to be mechanical in nature, with larvae selectively attacking the base of flowers, freeing petals from the receptacle and ultimately ‘trapping’ the anthers around the stigma as flowers approach anthesis. This phenomenon is different from floral galling, because male and female reproductive tissues remain viable, pistils develop into mature fruits, and viable seeds are produced. Floral galling typically involves deformed masses of tissues at the base of the corolla which house the florivore. This type of florivory results in abnormal development characterized by hypertrophy, hyperplasty, nutritive cell induction, and changes in floral primordial tissue^20–23^.

Florivore-initiated autogamy is also distinct from other herbivore-host interactions. In select cases (i.e. Fig wasps- Fig trees; Yucca moths- Yucca plants), ovipositing adult insects facilitate allogamy, which is ultimately beneficial to both herbivore and host^9,10^. Allogamy of *C. canadense* by *M. capella* would not be facilitated during oviposition, because adults are active and eggs are laid prior to anthesis early in floral development, it is unlikely this interaction might transition into or share specific characteristics of such ‘floral nursery’ systems^1^.

Florivore-initiated autogamy may be more widespread than currently recognized. Many species of *Mompha* are florivores, and may share adaptations facilitating autogamy in unopened flowers, driving evolution of floral morphology and herbivore resistance^24^. Several species of Onagraceae are parasitized by *Mompha* species, and these host-parasite systems have been proposed as models for testing evolutionary and ecological theory^2,25–27^. Larvae of yet-undescribed species of *Mompha* prefer large chasmogamous flowers of *Camissoniopsis cheiranthifolia* (Onagraceae). However, in this system, anthers are consumed, and flowers abscise before anthesis (a case of bud parasitism)^28^. Given the high number of *Mompha*-Onagraceae species interactions, this group may provide an excellent system to determine if our observations of florivore-initiated autogamy are more widespread^2,3,27–30^.

Florivores may provide significant selective pressure to influence plant and floral adaptations, and increased autogamy has been reported in other plants (e.g., *Fragaria spp*.) following florivory^29,30^. We propose feeding by *M. capella* may influence genetic diversity in *C. canadense* by two means. First, we demonstrate that autogamy in chasmogamous flowers is the direct result of florivory prior to anthesis. Second, we hypothesize such florivory will increase proportions of inbred seeds in a population already producing cleistogamous flowers, further reducing genetic diversity. Interactions reducing genetic diversity decrease survivability and adaptability of species, and will increase extinction risk in populations of species which are already endangered, disjunct, or which maintain relatively low diversity^3,25^. To test this hypothesis, future work should investigate the distributions of the host and the insect to determine if there are concurrent decreases in diversity in overlapping populations.

This study demonstrates, for the first time, selective florivory of floral structures which initiates autogamy and fruit-set in an unopened chasmogamous flower. This represents a new paradigm for insect-plant interactions along the spectrum from floral annihilation by florivores to pollination-mutualisms. Further study is required to determine consequences for individual fitness, persistence of populations, and broader implications of this newly documented interaction on the evolution of mating systems.

## Methods

We collected materials for this study on a weekly basis from June to August 2016, from populations on the 14 Wing Air Force base in Greenwood, NS (44.980726; −64.939513). For histological studies, flowering material at various stages of development was collected weekly or biweekly using an opportunistic sampling method; flowers were collected from visible plants at 5–10 m intervals while walking through populations. Flowers were collected in 50% Formalin Acetic Alcohol Fixative (5:5:90; Formalin: Acetic Acid: Ethanol). We fixed flowers for 48 hours and transferred them to 70% EtOH prior to dissection and microscopic analyses. Microscopic analyses included: (1) dissection and viewing/photography with an Olympus MVX-10 stereo zoom macroscope and Nikon Fi1 digital camera; (2) dissection, dehydration through a graded ethanol series prior to embedding in paraffin wax, followed by sectioning with an AO820 rotary microtome, adhering of sections to Poly-L-Lysine subbed glass slides, deparaffinization and staining with Safranin-O and Fast Green^31^, and viewing/photography with a Nikon Eclipse 50i bright field microscope and Nikon Fi1 digital camera; and (3) dissection, dehydration through a graded ethanol series; critical point drying in a Polaron E3000 Series Critical Point Drier (Quorum Technologies, East Sussex, UK) and mounting to aluminum stubs prior to gold-palladium coating with a SC7640 Sputter Coater (Quorum Technologies, East Sussex, UK), and viewing/photography in a JEOL JSM-5900LV Scanning electron microscope (JEOL, Peabody, MA, USA). All electronic images and photographic plates were processed and compiled using Photoshop CS6 (Adobe, Inc., San Jose, CA, USA).

Floral counts (chasmogamous and cleistogamous) were conducted during the chasmogamous blooming period, and at the end of the season. Individual chasmogamous flowers and cleistogamous flowers were dissected to quantify stamen number in early season, and seed number in late season. To quantify infestation rate of chasmogamous flowers, 30 unopened flowers were collected each week over a 4-week period in July (N = 120 collections). Infestation was recorded if there were *M. capella* larvae or frass present. Seed counts were performed by collecting mature fruit before opening (N = 120 fruit from each flower type). An unpaired two-tailed T-test with a Welch’s correction was used to test for significant differences in flower number, seed production, and seed germination (p < 0.05).

To mimic the effect of *Mompha capella* larvae severing petals and causing autogamous production of seeds, we used cyanoacrylate glue (Lepage’s Ultra Gel super glue) to keep petals from opening in 14 chasmogamous flowers. A small (2–3 mm) drop of glue was added to visible petal tips prior to anthesis. Flowers were visited weekly to observe that petals weren’t opened. After the end of chasmogamous flowering, glued flowers were collected and observed under a Nikon SMZ80 dissecting microscope for signs of autogamy and seed development.

Seed germination was tested on mature seeds collected from ripened chasmogamous (infested and uninfested) and cleistogamous fruit capsules throughout the season. After storage at 4 °C for 2–3 months, seeds were scarified using fine sandpaper and plated on Petri dishes lined with two sheets of filter paper moistened with 5 mL of reverse osmosis (RO) water. Plates containing 20 seeds per plate were covered and sealed within Ziploc bags. Seeds were incubated at 15 °C for a 12-hour photoperiod at low level light with an irradiance of 50 μmol m^−2^ s^−1^. Germinating seeds (with an emerged radicle) were counted and removed every 12 hours over a 2-week incubation period. Differences in germination rate were tested by One-Way ANOVA and means separated by Tukey’s multiple comparison test (p < 0.05).

All statistics were conducted as two-tailed tests, using GraphPad Prism version 7.00 for Mac, (GraphPad Software, La Jolla California USA).
